# Outbreak of Acute Gastroenteritis Among Rafters and Backpackers in the Backcountry of Grand Canyon National Park, April–June 2022

**DOI:** 10.15585/mmwr.mm7138a2

**Published:** 2022-09-23

**Authors:** Ariella P. Dale, Shanna Miko, Laura E. Calderwood, Ronan F. King, Matthew Maurer, Laurie Dyer, Marette Gebhardt, Wendy Maurer, Shawna Crosby, Mary E. Wikswo, Maria A. Said, Sara A. Mirza

**Affiliations:** ^1^Epidemic Intelligence Service, CDC; ^2^Arizona Department of Health Services; ^3^Maricopa County Department of Public Health, Phoenix, Arizona; ^4^Division of Viral Diseases, National Center for Immunization and Respiratory Diseases, CDC; ^5^Cherokee Nation Assurance, Tulsa, Oklahoma; ^6^National Park Service; ^7^Coconino County Health and Human Services, Flagstaff, Arizona.

On May 11, 2022, the National Park Service (NPS) Office of Public Health (OPH) and Coconino County Health and Human Services (CCHHS) in Flagstaff, Arizona contacted CDC about a rising number of acute gastroenteritis cases among backcountry visitors to Grand Canyon National Park (Grand Canyon). The agencies reviewed illness report forms, assessed infection prevention and control (IPC) practices, and distributed a detailed survey to river rafters and hikers with backcountry permits (backpackers) who visited the Grand Canyon backcountry. During April 1–June 17, a total of 191 rafters and 31 backpackers reported symptoms consistent with acute gastroenteritis. Specimens from portable toilets used by nine river rafting trip groups were tested using real-time reverse transcription–polymerase chain reaction and test results were positive for norovirus. Norovirus-associated acute gastroenteritis is highly transmissible in settings with close person-to-person contact and decreased access to hand hygiene, such as backpacking or rafting. IPC assessments led to recommendations for regular disinfection of potable water spigots throughout the backcountry, promotion of proper handwashing with soap and water when possible, and separation of ill persons from those who are not ill. Prevention and control of acute gastroenteritis outbreaks in the backcountry requires rapid reporting of illnesses, implementing IPC guidelines for commercial outfitters and river rafting launch points, and minimizing interactions among rafting groups. 

Commercially operated Colorado River rafting trips are allowed within the Grand Canyon during April–October (*1*). OPH surveillance of river rafting trip illnesses requires that guides on commercially operated trips report the occurrence of fewer than three illnesses at each trip’s end, contact the NPS by satellite phone as soon as possible when three or more illnesses occur (*2*), and complete an illness report form for each ill person. Private rafting trip guides must report illnesses within 7 days after completing the trip (*3*). Backpackers are encouraged to report illnesses.

During April–May 2022, approximately 4,770 rafters visited the Grand Canyon backcountry.^†^ On April 8, 2022, OPH was notified by a commercially operated rafting group within Grand Canyon of seven persons experiencing vomiting or diarrhea. After nine additional rafting trips (173 rafters), multiple cases of acute gastroenteritis were reported. OPH and CCHHS contacted CDC on May 11, 2022. By May 21, thirteen additional rafting trips with 102 reported cases of acute gastroenteritis were documented, and several backpackers reported symptoms consistent with acute gastroenteritis. A specific source of virus transmission had not been identified. On May 24, 2022, NPS requested CDC assistance, and an investigation was initiated. 

A case of acute gastroenteritis was defined as vomiting or diarrhea (at least three loose stools during a 24-hour period) <24 hours before trip launch through 3 days after the end of the trip in a person who participated in a river rafting trip or backcountry backpacking in the Grand Canyon during April 1–June 17, 2022. A detailed survey was distributed by email to all backpackers, river rafters on private and commercially operated trips with one or more ill persons, and river rafters on commercial trips with no reported ill persons during the same period. Survey responses were linked to illness report forms of previously reported illnesses to deduplicate. The survey closed on July 8, 2022. This activity was reviewed by CDC and was conducted consistent with applicable federal law and CDC policy.^§^

Among 116 illness report forms collected through July 8, 2022, a total of 94 (81%) rafters reported vomiting, 79 (68%) reported diarrhea, and 74 (64%) reported nausea. Acute onset, short symptom duration (median 24 hours), and predominance of vomiting suggested norovirus. CCHHS coordinated with the University of Arizona to test portable toilets for norovirus using real-time reverse transcription–polymerase chain reaction (*4*) with specimens from nine affected rafting trips and two unaffected trips. Pooled portable toilet specimens from each of the nine affected trips were positive for norovirus, including two specimens from river rafting trips that started in April 2022 (genotype 1) and seven specimens from river rafting trips that started in May 2022 (genotype 2). None of the pooled specimens from the portable toilets used during the two unaffected trips tested positive for norovirus. Portable toilet specimens were not tested for other pathogens. 

The date of first illness onset among rafters was April 6, 2022; the trip had an attack rate of 39% (11 of 28 rafters). Rafting trip attack rates ranged from 10% (three of 31) to 83% (29 of 35). During April 1–June 17, 2022, a total of 222 persons had an illness that met the case definition for acute gastroenteritis (Table) (Figure). Most respondents reported illness onset during the trip (178; 80%), with five persons from separate trips (two river rafters and three backpackers) reporting illness onset <24 hours before their trip started (different illness onset dates). Most cases occurred among park visitors (191; 86%) and the remaining cases (31; 14%) among professional guides.^¶^ Ill visitors were from 34 U.S. states and four additional countries. Among 222 acute gastroenteritis cases, 160 (72%) persons completed the electronic survey and provided sufficient information for further analysis (Table). Most (73%) illness onsets occurred during May 1–20, 2022. Survey response collection ended on July 8, 2022, with 1,327 visitors to the Grand Canyon backcountry completing at least a portion of the survey. Further analysis is underway to examine epidemiologic overlap among ill and non-ill rafters and backpackers who completed the survey.

**TABLE:** Characteristics of park visitors and guides with acute gastroenteritis (N = 222), by type of activity — Grand Canyon National Park, April 1–June 17, 2022

Characteristic	No. (%)		
	Commercial rafting trip*	Private rafting trip	Backpacking
**Persons with an illness report form or completed survey**			
**Total**	**136**	**55**	**31**
**Age, yrs, median, (IQR)**	55 (36–64)	39 (33–60)	40 (30–52)
**Gender**			
Female	65 (48)	20 (36)	12 (39)
Male	69 (51)	34 (62)	19 (61)
Nonbinary	1 (<1)	0 (—)	0 (—)
Did not specify	1 (<1)	1 (<1)	0 (—)
**Symptom onset**			
≤24 hrs before trip began	2 (<1)	0 (—)	3 (10)
During the trip	113 (83)	49 (89)	16 (52)
≤3 days after trip end	21 (15)	6 (11)	12 (39)
**National Park user type**			
Guide	30 (22)	0 (—)	1 (3)
Park visitor	106 (78)	55 (100)	30 (97)
**Persons who completed survey**			
**Total^†^**	**78**	**51**	**31**
**Age, yrs, median (IQR)**	57 (40–65)	39 (33–60)	40 (30–52)
**Race** **^§^**			
White	74 (95)	50 (98)	29 (94)
Asian, NH/OPI, or Other	3 (4)	0 (—)	2 (6)
Did not specify	1 (1)	1 (2)	0 (—)
**Ethnicity**			
Hispanic or Latino	1 (1)	1 (2)	3 (10)
Not Hispanic or Latino	73 (94)	46 (90)	27 (87)
Did not specify	4 (5)	4 (8)	1 (3)
**Symptom duration, median hours (IQR)**	24 (22–36)	24 (12–48)	24 (12–72)
**Reported interactions with persons from other trips**			
Yes	29 (37)	40 (78)	NA^¶^
No	49 (63)	11 (22)	NA
Did not specify	0 (—)	0 (—)	NA
Reported interactions with ill, suspected ill, or symptomatic persons**			
Yes	53 (68)	29 (57)	NA
No	25 (32)	22 (43)	NA
Did not specify	0 (—)	0 (—)	NA

**Abbreviations**: NA = not applicable; NH/OPI = Native Hawaiian or other Pacific Islander.

* Includes persons who completed an illness report form or electronic survey.

^†^ Includes only persons who completed electronic survey via email distribution.

^§^ None of the respondents identified as Black or African American or as American Indian or Alaska Native.

^¶^ Backpackers in the backcountry did not receive these questions.

** Reports of interactions with ill, suspected ill, or symptomatic persons might include persons on the same trip as the respondent.

**FIGURE:**
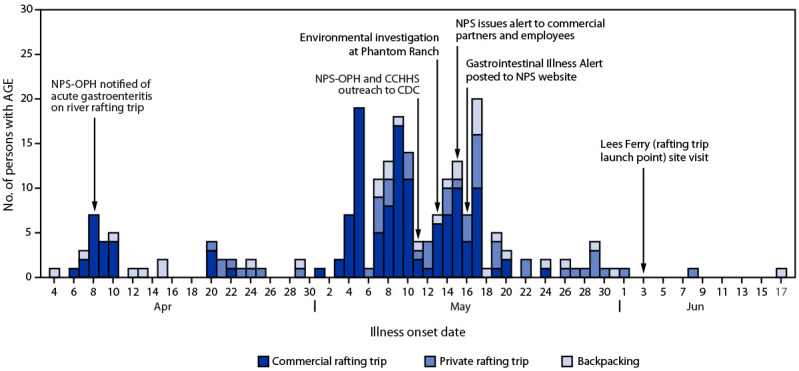
Number of persons with acute gastroenteritis among rafters and backpackers (N = 222*), by illness onset date — Grand Canyon National Park, April 1–June 17, 2022 **Abbreviations:** AGE = acute gastroenteritis; CCHHS = Coconino County Health and Human Services; NPS = National Park Service; NPS-OPH = National Park Service Office of Public Health. * Five rafters on private rafting trips were excluded because they reported insufficient information on date of illness onset.

Public health partners shared norovirus IPC education messages tailored to the backcountry environment immediately after notification (Figure). This included recommendations for symptom screening and exclusion of ill-persons from joining a rafting trip, disinfection of potable water, separation of ill persons from healthy persons, enhanced environmental cleaning, and strict precautions for food storage and preparation on river rafts in addition to environmental inspections of the commercial outfitters’ warehouses. OPH staff members conducted a site visit at Phantom Ranch** (a common exchange point) on May 13, 2022, and made recommendations for daily disinfection of the two potable water spigots using a chlorine solution and placement of mechanical backflow prevention devices between animal drinking trough hoses and potable water supply hoses. Frequent communication occurred among commercial outfitters, the backcountry office, and public health agencies to expedite information exchange, including the sharing of portable toilet test results. 

NPS posted multiple acute gastroenteritis website alerts^††^ to provide prevention education beginning on May 16, 2022, including a link to CDC’s *Norovirus* and *Safe Drinking Water *webpages.^§§^ Outfitter staff members were advised to promote handwashing with soap and water, monitor adherence, and isolate or cohort persons with acute gastroenteritis during the trip whenever possible. Many outfitter staff members were unaware that alcohol-based hand sanitizer is ineffective in mitigating norovirus transmission (*5*). OPH and CDC conducted a site visit to the Lees Ferry raft launch point on June 3, 2022 and recommended adding signs to promote handwashing in restrooms, displaying acute gastroenteritis outbreak information on bulletin boards throughout the backcountry, and increasing the frequency of cleaning restrooms and disinfecting the potable water spigot, a highly used water source by rafters and day visitors.

## Discussion

A large norovirus-associated outbreak of acute gastroenteritis occurred in the Grand Canyon backcountry among river rafters and backpackers during April–June 2022. Preliminary analyses of illness characteristics and portable toilet specimen test results suggested norovirus as the primary causative agent of illness. Norovirus spreads quickly through person-to-person contact and contaminated food or beverages, and can persist in the environment (*5*). Five persons reported illness onset <24 hours before their trips were launched and two genotypes were identified from portable toilet specimens of affected trips, indicating a potential for multisource introduction of norovirus into the river corridor. Analyses of survey responses are underway to identify epidemiologic overlap, including food and beverages, river stop locations, backcountry toilet use, and other factors.

Illness reports slowed before the arrival of the CDC team on May 31, 2022. The close relationship among outfitters and public health authorities likely facilitated rapid communication about the rise in acute gastroenteritis cases that resulted in more vigilant warnings during pretrip passenger briefings and an internal reinforcement of environmental protection and equipment sanitation guidelines (*2*). The last report of acute gastroenteritis occurred on June 17, 2022.

The findings in this report are subject to at least two limitations. First, although no individual specimens were available for testing, test results from pooled portable toilets suggest norovirus as a primary contributor to this outbreak. Second, the total number of illnesses associated with this outbreak is likely underreported. OPH has adapted sanitation standards and IPC recommendations to meet the unique setting of river rafting and backcountry camping trips. Some acute gastroenteritis, including norovirus, is expected on rafting and hiking trips (*6*). Norovirus is highly infectious and has a low infective dose (*5*). Because many trips use the same campsites and place portable toilets in the same locations, particles could have been transmitted to surfaces, beach sand, or river water where new groups could have encountered them, and then transmitted the virus both from person-to-person and trip-to-trip. Rapid separation of ill persons from non-ill persons and reinforcement of hygiene and sanitation practices by commercial rafting trip guides might have led to lower attack rates reported on some trips. 

Previous norovirus outbreaks have occurred among river rafters in Grand Canyon associated with contaminated food products (*7*) and person-to-person transmission (*8*) resulting in recommendations to adhere to strict hygiene guidance. An increase in norovirus activity was observed at a national level in spring 2022, with the number of outbreak reports returning to prepandemic levels for the first time since March 2020 (*9*). 

With norovirus increasing nationwide and visitation rates returning to near prepandemic levels (*10*), the potential exists for resurgence of norovirus outbreaks among visitors to the Grand Canyon backcountry. River rafting and camping might amplify norovirus spread because of limited hygiene supplies and close person-to-person contact. Prevention and control of future outbreaks includes rapid reporting of illnesses, symptom screening before trip launch to minimize introduction of illnesses, strict adherence to hand hygiene with soap and water and sanitation protocols, disinfection of water before consumption, prompt separation of ill passengers, and minimizing of interactions with other rafting groups.

**Summary**

**What is already known about this topic?**

Norovirus-associated acute gastroenteritis is highly transmissible in settings with close person-to-person contact and decreased access to hand hygiene, such as backpacking or rafting.

**What is added by this report?**

During April 1–June 17, 2022, the largest outbreak of acute gastroenteritis documented in the Grand Canyon National Park backcountry occurred. At least 222 rafters and backpackers became infected, probably with norovirus. Strong partnerships with river outfitters and National Park staff members enabled implementation of prevention and control measures.

**What are the implications for public health practice?**

Outbreak control measures in the setting of rafting and backpacking include rapid case reporting, symptom screening before trip start, water disinfection, prompt separation of ill passengers, strict adherence to hand hygiene with soap and water, and minimizing interactions among rafting groups.

## Footnotes

* These authors contributed equally to this report.

† https://grcariverpermits.nps.gov/viewRiverCalendars.cfm (Accessed September 20, 2022).

§ 45 C.F.R. part 46, 21 C.F.R. part 56; 42 U.S.C. Sect. 241(d); 5 U.S.C. Sect. 552a; 44 U.S.C. Sect. 3501 et seq.

¶ All cases among guides occurred after illness onset among rafters on the same trip.

**
https://www.nps.gov/grca/learn/photosmultimedia/grand-canyon-in-depth-03.htm

††
https://www.nps.gov/grca/planyourvisit/conditions.htm

§§
https://www.cdc.gov/healthywater/drinking/travel/index.html
